# Fear of progression in Parkinson's disease: Role of age and occupational status

**DOI:** 10.1177/1877718X251365243

**Published:** 2025-11-18

**Authors:** Simone D’Souza, Esther Tekampe, Björn Falkenburger, Nils Schnalke

**Affiliations:** 1 Department of Neurology, University Hospital Carl Gustav Carus Dresden at TU Dresden, Dresden, Germany; 2 Department of Psychiatry, Psychosomatic Medicine and Psychotherapy, Goethe University Frankfurt, University Hospital Frankfurt, Frankfurt, Germany; 3 German Center for Neurodegenerative Diseases, Deutsches Zentrum für Neurodegenerative Erkrankungen (DZNE), Standort Dresden, Dresden, Germany

**Keywords:** Parkinson's disease, non-motor symptoms, fear of progression, distress, depression, anxiety

## Abstract

**Background:** Parkinson's disease (PD) is a slowly progressing neurodegenerative disorder, so it is likely that people with PD (PwPD) face increasing disability. PwPD thus experience various degrees of fear of progression (FoP), which can become dysfunctional. **Objective:** This study aims to examine the prevalence of and contributing factors to dysfunctional FoP in PwPD. **Methods:** The Fear of Progression Questionnaire–Short Form (FoP-Q-SF) was administered along with further questionnaires for non-motor symptoms; PD motor symptoms as reported by the Unified Parkinson's Disease Rating Scale Part III (UPDRS III) were obtained from patient charts. **Results:** 28% of the 105 PwPD (mean age 66 years, 56% Hoehn & Yahr stage I/II) were categorized as experiencing dysfunctional levels of FoP using the established cut-off score of ≥34. Our analyses revealed that the FoP-Q-SF underestimates the prevalence of dysfunctional FoP in older and non-working PwPD. Using a more appropriate cut-off, 33% of PwPD are classified as having dysfunctional levels of FoP. We found strong correlations of FoP with measures of anxiety, depression and quality of life. Disease duration was secondary to these factors. We found no associations between FoP and motor symptoms. **Conclusions:** Our findings confirm that dysfunctional FoP significantly impacts the psychological well-being of PwPD, affecting one in three PwPD and contributing to heightened anxiety, depression, and reduced quality of life. Future validation studies are needed to confirm the cut-off value proposed here and to enable a better integration of the concept of FoP into routine care for PwPD.

## Plain language summary

This study investigates the prevalence and the factors affecting fear of progression (FoP) in people with Parkinson's disease (PwPD). FoP refers to concerns of worsening symptoms of PD over time, which can be rational and even useful, but also negatively affect well-being when FoP becomes dysfunctional. We used the Fear of Progression Questionnaire–Short Form (FoP-Q-SF) among other questionnaires to assess motor and non-motor symptoms of PD.

Among the 105 PwPD in our study, 28% were characterized as having dysfunctional FoP when using the standard cut-off score. We found that the questionnaire used to determine which PwPD experience dysfunctional amounts of FoP underestimates FoP in older and non-working people. After adjusting the cut-off to account for this, 33% of PwPD were characterized as experiencing dysfunctional amounts of FoP. Furthermore, more FoP was related to more depressive symptoms, more anxiety and poorer quality of life. The relationship to these psychological factors was stronger than the relationship to the duration of PD or the severity of motor symptoms.

In summary, one in three PwPD struggle with dysfunctional amounts of FoP, which impacts their well-being. To better detect dysfunctional FoP in PwPD, we suggest using a cut-off when screening PwPD for FoP that is different from the one used in other diseases. Further studies are needed to substantiate our findings and to enable the better integration of FoP assessments into routine PD care.

## Introduction

Parkinson's disease (PD) is a chronic and slowly progressing disease.^1^ Even though dopamine-dependent motor symptoms are readily treatable, the emergence of symptoms that are not primarily caused by a lack of dopamine—such as impaired balance or cognition—eventually necessitate at least some degree of dependence on others. A confrontation with the loss of one's autonomy can be rational and useful.^2^ Nevertheless, these symptoms, and specifically the dependence they cause, typically occur in the latest stage of PD.^3^ Hence, rational worries about one's future may turn into fear and become dysfunctional. This phenomenon has been termed “fear of progression” (FoP) or health anxiety.^2^ Currently, there are only two studies concerned with FoP in people with Parkinson's disease (PwPD). Berg and colleagues compared FoP in several chronic conditions and found that the extent of FoP experienced by PwPD is second only to rheumatologic diseases.^4^ In 2022, Folkerts and colleagues published preliminary data from a multi-center study in PwPD (n = 120) using the Fear of Progression-Questionnaire (FoP-Q) among other psychometric scales and assessments. In their study, they found that 80% of PwPD had moderate to dysfunctional FoP, describing higher levels of FoP in women and in younger patients and in PwPD with cognitive dysfunction.^5^

In the same regard, many PwPD suffer from psychiatric conditions, especially distress, anxiety and depression.^6^ The incidence of the latter is estimated to be around 40% in PwPD,^7^ underlining the possibility of a shared etiology and the potential benefits of adequate counselling, which relates to psychotherapeutic treatment and patient education, including the education of those caring for PwPD.

Using data from a cross-sectional study of PwPD,^6^ we examined whether the short form of the fear of progression questionnaire (FoP-Q-SF) constitutes an adequate means to measure FoP in PwPD. Furthermore, we examined how it relates to disease-specific features, especially to non-motor symptoms like distress, anxiety and depression. In addition, we tried to identify patient characteristics associated with higher levels of FoP.

## Methods

### Participants

The data presented here are part of a previously published study, which aimed to validate the Distress Thermometer for Parkinson's Disease.^6^ The observational cross-sectional study was conducted at the Department of Neurology at the University Hospital “Carl Gustav Carus” in Dresden, Germany, between January 2019 and October 2020. Inclusion criteria were (1) diagnosis of PD, (2) sufficient cognitive ability to complete the questionnaires and (3) proficiency of written and spoken German. All inclusion criteria were assessed by the treating movement disorders specialist. PD diagnosis was confirmed by the same physician.

The study was approved by the ethics committee at Technische Universität Dresden (IRB00001473, EK 37012019) and conducted in accordance with relevant guidelines and regulations. We confirm that we have read the Journal's position on issues involved in ethical publication and affirm that this work is consistent with those guidelines. All patients gave written informed consent to participate in the study.

**Figure 1.**
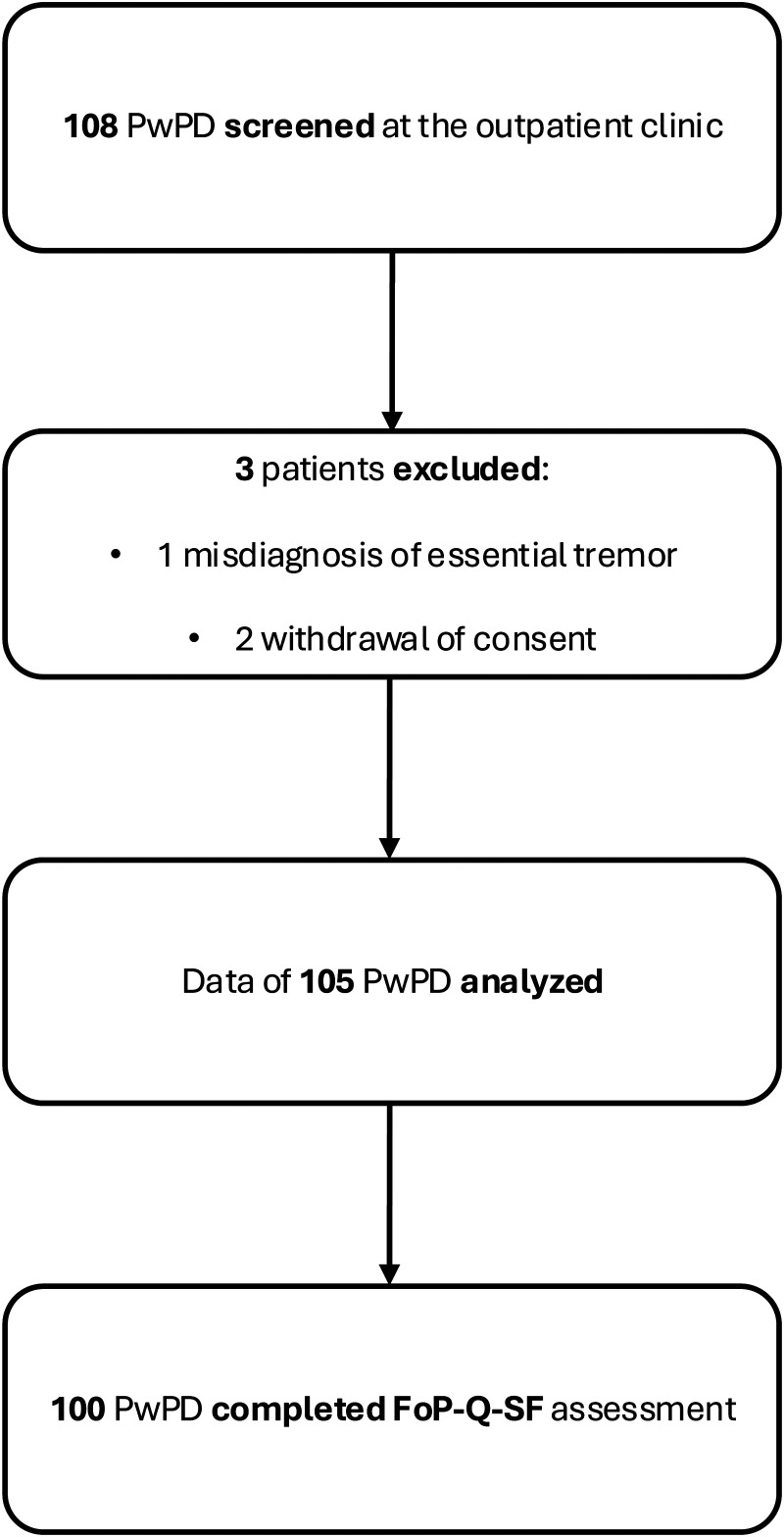
Flow chart of study participants. PwPD: people with Parkinson's disease, FoP-Q-SF: Fear of Progression Questionnaire – Short Form.

One-hundred and eight consecutive patients with PD were enrolled in this study. They were recruited in the waiting area of the PD outpatient clinic, where they waited for their regular follow-up appointment. Three participants were excluded. One participant was excluded as they did not have PD and two withdrew from the study. The data of 105 participants were analyzed and 100 PwPD completed the FoP-Q-SF assessment (Figure 1).

### Assessments

Patients were provided with the FoP-Q-SF. The FoP-Q-SF is a questionnaire with 12 items on a 5-point Likert scale. The scores thus range from 12 to 60 and higher scores indicate more fear of progression. The FoP-Q-SF is described as having a one-factor structure.^8,9^ The FoP-Q-SF has been validated in patients with arthritis, malignant diseases and in spouses of chronically ill patients and a cut-off of ≥ 34 points was found to indicate dysfunctional levels of FoP.^8,10,11^

For some analyses, we excluded questions 4 and 12 of the FoP-SF-Q and recalculated the cut-off score for FoP as follows: The established cut-off of 34/60 points corresponds to 57%, which equates to 30 of 52 points when excluding questions 4 and 12.

Along with the FoP-Q-SF, participants were provided with the Distress Thermometer (DT) for Parkinson's Disease, the Hospital Anxiety and Depression Scale (HADS), the Patient Health Questionnaire-9 (PHQ-9), the Non-motor Symptom Questionnaire (NMSQ), the Generalized Anxiety Disorder Scale-7 (GAD-7), the Herth Hope Index (HHI), the Schedule for the Evaluation of Individual Quality of Life (SEIQoL) and a demographic survey as paper-based questionnaires. The demographic survey included gender, age, marital status, education, occupational status, disease related questions (such as duration of disease or medication) and questions about supportive therapies, past and present psychological counselling and coping strategies.

PD motor-subtype, Hoehn &Yahr (H&Y)-stage and Unified Parkinson's Disease Rating Scale Part III (UPDRS III, assessed in MED ON) total scores were collected from the routine clinical assessment of the same day as the outpatient clinic visit. German versions were used for all questionnaires.

### Statistical analyses

Statistical analyses were performed with JASP and python. To assess differences between two groups, a Chi-square test was used for categorical variables, a Mann-Whitney *U* test for metric and non-normally distributed variables, a *T*-test for metric, normally distributed variables, and Fisher's exact test for small sample sizes. Correlations between different parameters were calculated using Spearman's rank correlation or Pearson's r where applicable. Multiple testing was corrected for using Bonferroni's method.

An exploratory factor analysis was conducted to determine whether the items of the FoP-Q-SF loaded onto only one factor in our cohort of PD patients. To assess which patient characteristics influence FoP as measured by the FoP-Q-SF, we conducted a random forest regression. As an alternate mean of calculating suprathreshold FoP, we utilized the mean +/- one SD to determine which patients have suprathreshold FoP. For this, mean scores of the FoP-Q-SF were converted to Z-scores and thus, a Z-score of −1 to 1 indicates moderate FoP, a Z-score of < -1 indicates low levels of FoP and a Z-score of >1 indicates high levels of FoP. This is analogous to the methodology used by Folkerts and colleagues.^5^ The sample size was calculated based on the validation of a different assessment. Still, as generally a subject to item ratio of at least five (i.e., in this case 5 × 12 questions of the FoP-Q-SF = 60) is recommended for exploratory factor analysis, the sample size in this case can be considered appropriate.^12^

## Results

One-hundred and five patients were enrolled in this study. Demographic and clinical characteristics of the participants are listed in Table 1. The single items of the FoP-Q-SF and their respective means and standard deviations (SD) are displayed in Table 2.

**Table 1.** Demographic and clinical characteristics.

Feature	N = 105	Mean (SD) or %
Gender, % male	68	65
Age (years)	102	66 (Range 35–87)
*PD subtype*		
Equivalent - type	31	29.5
Tremor - dominant type	25	23.8
Hypokinetic - rigid type	46	43.8
NOS	2	1.9
Missing	1	1
*Hoehn & Yahr stage*		
Hoehn & Yahr stage I	8	7.6
Hoehn & Yahr stage II	51	48.6
Hoehn & Yahr stage III	38	36.2
Hoehn & Yahr stage IV	5	4.8
Hoehn & Yahr stage V	0	0
Missing	3	2.9
*UPDRS III total score*	103	22.4 (10.3)
*Disease duration [years]*	105	8.9 (5.7)
*Secondary diagnoses*		
MCI	4	3.8
Dementia	6	5.7
Depression	18	17.1
*Medication*		
Levodopa	84	80
Dopamine agonist	78	74.3
COMT inhibitor	34	32.4
NMDA antagonist	14	13.3
MAO-B inhibitor	47	44.8
*Marital status*		
Single	5	4.8
Married	82	87.1
Widowed	6	5.7
Divorced	10	9.5
Missing	2	1.9
*Education*		
School (≥ 10 years)	45	42.9
School (< 10 years)	19	18.1
University	36	34.3
Missing	5	4.8
*Occupational status*		
Working	27	25.7
Not working	77	73.3
Missing	1	1

SD: standard deviation, PD: Parkinson's disease, NOS: not otherwise specified, MCI: mild cognitive impairment, COMT: catechol-o-methyl-transferase, NMDA: N-methyl-d-aspartate, MAO-B: monoamine oxidase B.

**Table 2.** Mean values of the fear of progression questionnaire – short form (FoP-Q-SF).

Item	Mean	SD
All items	2.47	0.48
1. Being afraid of disease progression	2.87	1.04
2. Being nervous prior to appointments with physicians or periodic examinations	2.13	1.09
3. Being afraid of pain	2.32	0.95
4. Being afraid of becoming less productive at work	1.70	1.16
5. Having physical symptoms (e.g., rapid heartbeat, stomachache)	2.46	1.03
6. Being afraid of the possibility that the children could contract the disease	2.55	1.30
7. Being afraid of relying on strangers for activities of daily living	3.12	1.21
8. Being afraid of no longer being able to pursue hobbies	2.96	1.30
9. Being afraid of severe medical treatments in course of illness	2.62	1.06
10. Worrying that medications could damage the body	2.77	1.15
11. Worrying what will become of family if something happens to me	2.58	1.08
12. Being afraid of not being able to work anymore	1.54	1.04
Total score	29.48	6.98

SD: standard deviation.

We noted that the mean response to questions 4 and 12 differed from the other items of the FoP-Q-SF (Table 2, question 4 mean 1.7 ± 1.1.59, question 12 mean 1.545 ± 1.044, all other items: mean 2.613 ± 0.636, paired T-test between Q4, Q12 and mean of all other variables p < 0.001). These questions concern the fear of losing one's occupation. Patients in our study were 66 ± 10 years old and only 27% of patients indicated that they were still working. The FoP-Q-SF is reported to load on only one factor.^8^ In our dataset, however, an Exploratory Factor Analysis (EFA) revealed two factors with Q4 and Q12 loading on a separate factor. Indeed, the responses to questions 4 and 12 were highly correlated (Spearman's rho, r = 0.628, p < 0.001), and the EFA revealed one factor when excluding questions 4 and 12 (Bartlett's test p < 0.001, Chi-square test p < 0.001, r^2^ = 0.357). Internal consistency of the entire FoP-Q-SF was acceptable with a Cronbach's alpha of 0.774 ± 0.06 and increased to 0.784 ± 0.07 when excluding questions 4 and 12.

The FoP-Q-SF has been established in diseases typically affecting individuals that are younger than our cohort and with a higher proportion of working individuals. The established cut-off score of >34 points identified 28% of PwPD with suprathreshold FoP in our study. Previous studies that have validated the FoP-Q-SF in different conditions mainly focus on its psychometric properties and not on reporting rates of FoP.^8,9,11,13–15^ One study in patients with alveolar echinococcosis found a similar prevalence of FoP of 38%,^16^ whereas a study in carriers of mutations in the mitochondrial genome with variable expression found a lower prevalence of 18%.

Considering the demographic characteristics, we then compared the level of FoP with respect to age, sex, social status and level of education, finding no significant correlation with the total FoP-Q-SF score (irrespective of omitting or incorporating questions 4 and 12). There were no significant differences between men and women concerning the prevalence of suprathreshold FoP (Mann-Whitney U, p = 0.087). In addition, we analyzed the FoP-Q-SF without questions 4 and 12, using an adapted cut-off score of 30 points. This analysis identified 29% of PwPD with suprathreshold FoP in the total cohort. Among the 73% of PwPD who were not actively working anymore, 33% were classified as having suprathreshold FoP. Among the 27% of PwPD still in the workforce, 15% were classified as having suprathreshold FoP when questions 4 and 12 were omitted (cut-off 30 points) whereas 33% of occupied PwPD were classified as having suprathreshold FoP when all items of the scale were included (Chi-square test p < 0.001). These differences suggest that questions 4 and 12 should not be excluded for patients who are still in the workforce.

As an alternative approach, we applied cut-off scores that are based on the mean of the cohort as described by Folkerts et al.,^5^ defining z-scores ≤ -1 as subthreshold, −1 < z ≤ 1 as moderate FoP and z ≥ 1 as dysfunctional FoP. With this classification, 85% of PwPD in our cohort experienced at least moderate amounts of FoP. When excluding Q4 and Q12, the proportion of PwPD with at least moderate FoP increased to 88%. These numbers are comparable with those obtained by Folkerts et al.^5^ Hence, FoP is a matter concerning the vast majority of PwPD.

56.6% of patients reported that they currently have a need for psychosocial counselling. Of the 43.4% of patients who are currently not in need of counselling, 57% would have needed psychological support when the diagnosis of PD was made. 14.3% of PwPD in this study currently underwent psychotherapeutic treatment and 20% were in psychotherapeutic treatment previously. PwPD currently undergoing psychotherapy indicated higher levels of FoP (Mann-Whitney-U, p = 0.023, excluding Q4 and Q12), but those that underwent psychotherapy in the past did not indicate more FoP than those who never received psychotherapy (Mann-Whitney-U, p = 0.28). 49.5% of PwPD in our study expressed a need to be able to consult their physician about psychosocial topics and only 17% of PwPD are in contact with other PwPD as a coping strategy.

When analyzing the degree of FoP with respect to disease duration and comparing participants with early PD (≤ five years) to those with a disease duration of > five years, more patients with a shorter disease duration were classified as having suprathreshold FoP (40.5% in ≤ five years, 20.6% in > five years, Chi-square test, p = 0.032). Without questions 4 and 12, 34.3% of patients with early PD and 25.8% of patients with more advanced PD were classified as having suprathreshold FoP, but the Chi-square test was no longer significant (p = 0.38).

We found strong correlations between the extent of FoP and measures of anxiety and non-motor symptoms, namely HADS-A (Spearman's rho, r = 0.612, p < 0.001), GAD-7 (r = 0.55, p < 0.001) and NMSQ (r = 0.516, p < 0.001). There was a strong negative correlation with hope as measured by the HHI (r = -0.511, p < 0.001) and a moderate correlation with distress as measured by the Distress Thermometer (r = 0.299, p = 0.003). We found no significant correlations with UPDRS, disease duration or age at onset and only a weak correlation with Hoehn and Yahr-stage (r = 0.21, p = 0.042) and a weak negative correlation with quality of life as measured by the SeiQoL (r = -0.257, p = 0.002). There were no significant correlations between disease duration or severity of motor symptoms with FoP even when conducting the correlation analyses separately for earlier or more advanced PwPD. Single items of the SeiQol correlated more strongly with FoP, the strongest correlations were found between one's satisfaction with one's own physical health (r = -0.328, p < 0.001) and one's emotional well-being (r = -0.437, p < 0.001). With respect to subjective cognitive problems, PwPD who indicated having problems with memory (Q12 of the NMSQ) or with attention (Q15) also indicated more FoP (27.6 vs. 25.0, p = 0.044 for Q12, 29.1 vs. 24.1 for Q15, p < 0.001, Student's t-test). There was no significant difference in age or disease duration between these groups. As our analyses indicated better internal consistency and better sensitivity when omitting questions 4 and 12 from the FoP-Q-SF, these analyses were conducted without questions 4 and 12.

To find factors that are associated with an increase in FoP, we tried to predict FoP-Q-scores (excluding questions 4 and 12) from patient characteristics using a random forest regression. The best performing model encompassed: measures of anxiety (HADS-A, mean decrease in accuracy [MDA] = 6.33 and GAD-7, MDA = 3.75), depression (HADS-D, MDA = 2.89), of hope (HHI, MDA = 5.85), non-motor symptoms (NMS-Quest, MDA = 4.74)) and disease duration (MDA = 0.86), the latter being the least important model feature. This model was able to predict FoP-Q-SF-scores with a mean absolute error of 3.27, a mean absolute prediction error of 15.2% and an r^2^ of 0.51, i.e., the model explained 51% of the variance of FoP-Q-SF-scores.

## Discussion

In this study, we applied the FoP-Q-SF to measure FoP in PwPD. We found significant effects of work status on the prevalence of suprathreshold FoP and effects of anxiety, depression, hope, and non-motor symptom burden on the extent of FoP in PwPD.

When using the 34-point cut-off for FoP established for other diseases,^8,10,11^ the FoP-Q-SF tends to underestimate the prevalence of FoP in our elderly cohort, as two of the twelve questions (Q4 and Q12) relate to one's occupation and only 27% of patients in our cohort were still working. Specifically, 27% of the PwPD in our study were classified as FoP with the 34-point cut-off, which is lower than the 33% obtained for study participants that still are in the work force (34-point cut-off) and to 33% obtained for study participants that no longer work when excluding questions 4 and 12 and applying an adapted 30-point cut-off.

The importance of work status for the analysis of the questionnaire is underlined by the results of the exploratory factor analysis showing that the FoP-Q-SF loads onto two separate factors in our cohort as opposed to one as described in the initial validation study.^8^ This discrepancy is probably explained by the fact that the original questionnaire was validated in younger patients (patients with chronic arthritis and malignant diseases)^11^ and thus in a population with higher rates of employment.

Excluding Q4 and Q12 increased the internal consistency of the FoP-Q-SF in our cohort. Yet, excluding questions 4 and 12 would underestimate the prevalence of FoP in working PwPD. The FoP-Q-SF could thus be modified to include a dichotomous question such as “Are you still actively working?”. If the answer to this question is “no”, then a cut-off of ≥30 could be applied. If the answer is “yes”, the validated cut-off of ≥34 could be applied. Our 30-points cut-off for the FoP-Q-SF without questions 4 and 12 was chosen proportionally to the 34-point cut-off. The 34-point cut-off was chosen based on the median of the sample's total FoP-Q-SF score.^11^ The median score with questions 4 and 12 excluded in our sample was 29, which could constitute an alternative cut-off for participants who are no longer in the work force. Further validation studies are needed to validate these proposals in a cohort of PwPD.

Thoughts or worries about increasing dependency with disease progression are rational and can be helpful for patients and caregivers, e.g., by establishing additional care or initiating apartment remodeling. FoP thus may contribute to prolonged autonomy. When these thoughts turn into fear, however, they can become dysfunctional and hamper coping. This is exemplified by the fact that PwPD with more FoP experience more anxiety and depressive symptoms (HADS-A and -D, GAD-7), have less hope (HHI) and experience more non-motor symptoms (NMS-Quest). This association is also evident in other conditions.^17^

As evidenced by random forest regression, disease duration only plays a minor role in FoP, and the extent of motor symptoms (UPDRS III and H&Y-stage) was not robustly associated with FoP. Fittingly, only the *subjective* satisfaction with one's physical and mental health and emotional well-being was associated with the extent of FoP. However, the cross-sectional design of our study limits our ability to draw conclusions as to whether more severe motor symptoms or a longer disease duration are associated with heightened levels of FoP, as interindividual differences likely underlie the observed associations. To determine the effect of disease progression on FoP, longitudinal studies assessing the intraindividual course of FoP within the same PwPD are required.

Moreover, cognitive ability was not explicitly assessed as a part of this study, and cognitive impairment or anosognosia might have obscured an association between disease duration and FoP, as higher anosognosia is related to a lesser awareness of non-motor symptoms in PD.^18,19^ In line with this, PwPD in our study with subjective cognitive problems (i.e., without anosognosia) indicated more FoP, which could point towards an inverse relationship between anosognosia and FoP. Nevertheless, as less than 10% of PwPD in our cohort were diagnosed with either MCI or PDD, it seems unlikely that cognitive impairment is the primary explanation for the lack of association observed between disease duration, motor symptoms and FoP. Involving the primary caregiver (e.g., through a separate questionnaire) could have been useful to further investigate a potential relationship between cognitive symptoms, anosognosia and FoP.

PwPD with higher FoP tend to seek psychotherapy more often and those PwPD who had psychotherapy in the past indicated less FoP. This exemplifies that psychological counselling increases coping abilities as has been shown in cancer patients.^11^ Unfortunately, the supply of psychological counselling does not match the demand: Half of PwPD in our study indicated a need for psychological counselling. In addition, half of those who did not currently feel a need for psychological counselling wished for counselling when the diagnosis of PD was made. Half of PwPD in our study also expressed the need for a consultation with their neurologists about psychosocial topics, which might indeed be a way to at least partly mitigate dysfunctional FoP. People knowledgeable about PD—members of the therapeutic team such as physicians, physiotherapists, psychotherapists, but also other PwPD, or partners of PwPD—could help PwPD in striking a balance between rational thoughts and measures related to one's future, which undoubtedly changes with a diagnosis of PD, and dysfunctional FoP, which likely does not serve any benefit.

There are limitations to this study. More than half of the patients belonged to Hoehn & Yahr stage I/II, which is associated with a lower disease burden. Motor symptoms were not robustly associated with FoP in our study, but this could be a sampling bias as discussed above. Second, this is a cross-sectional study at a single center and thus, the conclusions that can be drawn from this study are limited. We also cannot draw any conclusions pertaining to a comparison of the FoP-Q-SF and its relation to its longer counterpart, FoP-Q. Third, we used the SeiQoL to reduce patient-burden as it contains less questions than the more PD-specific Parkinson's Disease Quality of Life Questionnaire (PDQ-39), limiting comparability with other studies. The shorter PDQ-8 might have been a useful alternative in this case. Fourth, cut-off scores for patients who are no longer in the workforce need to be established for the FoP-Q-SF in general, independent of PD diagnosis.

In conclusion, our findings confirm that FoP significantly impacts the psychological well-being of PwPD, affecting one in three PwPD and contributing to heightened anxiety, depression, and reduced quality of life. Future validation studies are needed to confirm the cut-off values to enable a better integration of the FoP concept into routine care for PwPD.
